# Response of Various Yb^3+^-Doped Oxide Glasses to Different Radiation Treatments

**DOI:** 10.3390/ma15093162

**Published:** 2022-04-27

**Authors:** Mikko Hongisto, Sylvain Danto, Marian Ghena, Decebal Iancu, Daniel Ighigeanu, Laura Mihai, Véronique Jubera, Laeticia Petit

**Affiliations:** 1 Photonics Laboratory, Tampere University, Korkeakoulunkatu 3, 33720 Tampere, Finland; laeticia.petit@tuni.fi; 2 CNRS, Bordeaux INP, Institut de Chimie de la Matière Condensée de Bordeaux (ICMCB), UMR 5026, University Bordeaux, F-33600 Pessac, France; sylvain.danto@u-bordeaux.fr (S.D.); veronique.jubera@u-bordeaux.fr (V.J.); 3 Linear Accelerators Laboratory, National Institute for Laser, Plasma and Radiation Physics, 077125 Magurele, Romania; marian.ghena@inflpr.ro (M.G.); daniel.ighigeanu@inflpr.ro (D.I.); laura.mihai@inflpr.ro (L.M.); 4 Horia Hulubei National Institute of Physics and Nuclear Engineering, 077125 Magurele, Romania; decebal.iancu@nipne.ro

**Keywords:** phosphate, borosilicate, germanate, tellurite, glass, irradiation, ytterbium

## Abstract

The radiation effects of electrons and protons on the spectroscopic and optical properties of oxide glasses doped with Yb^3+^ in various glass systems were investigated to understand the impact of the glass composition on the glass photo-response. Changes in the optical and emission properties were seen after the radiation treatment, and the magnitude of the changes depended on the irradiation source and dose. For all the investigated materials, the absorption coefficients in the 200–550 nm range increase post-irradiation, revealing the formation of defects in the glasses during the irradiation. While the spectroscopic properties of the tellurite glass remain unchanged, a small reduction in the Yb^3+^ emission intensity was seen after irradiating the phosphate, borosilicate, and germanate glasses, indicating that a reduction of Yb^3+^ to Yb^2+^ might occur in these glasses during the radiation treatment. The changes in the optical and spectroscopic properties after proton irradiation are small as they are localized at the surface of the glasses due to the shallow penetration depth of the proton in the glass. Even though the doses are small, the electron irradiation produces larger changes in the optical and spectroscopic properties since the electrons penetrate the entire volume of the glasses. All the changes in the optical and spectroscopic properties of the glasses were successfully reversed after a short heat treatment revealing the reversible nature of the photo-response of the investigated glasses.

## 1. Introduction

For many decades now, trivalent rare-earth (RE^3+^) doped glasses have been the subject of many studies due to their optical and spectroscopic properties, especially the glasses doped with Yb^3+^ ion due to its simple band structure, which is useful for signal amplification in the ~1.06 µm band. Especially, the Yb^3+^ doped silica fibers have found broad acceptance in the industry, defense, life sciences, etc. [1] thanks to their important assets spanning great efficiency, beam quality, and limited upkeep costs. 

However, the laser performance of such fibers has been reported to be affected by the gradual increase in the losses during laser operation. This phenomenon is known as the photodarkening process [2]. The high-intensity light ionizes the silica glass matrix, releasing electrons, breaking bonds in the matrix, and forming color centers [3]. These centers absorb light, mainly in the UV-visible range but with some absorption extending to laser wavelengths, and are thought to reduce the fiber performance during laser operation. Despite the large number of studies devoted to the photodarkening phenomenon in silica fiber [4,5,6], the mechanism of photodarkening in Yb-doped silica is still under debate and has not yet been confirmed. Nonetheless, silica fibers with minimized photodarkening have been successfully developed. For example, the addition of P_2_O_5_ and Al_2_O_3_ in the Yb^3+^ doped silica glass was reported to suppress photodarkening due to the presence of AlPO_4_ units in the silica network, which increased the Yb^3+^ion solubility in the silica network [7,8]. These studies clearly show the crucial influence of the glass composition on the photo-response of silica glass. 

One should point out that most of the studies related to the photo-response of Yb^3+^ doped glasses have been focused on silica glass, although other glass systems are also good glass hosts for Yb^3+^ ions. For example, phosphate glasses doped with Yb^3+^ are potential materials for the generation of ultrashort pulses and tunable laser sources due to their broad absorption and emission bands and high absorption/emission cross-section [9]. Yb^3+^ doped germanate and tellurite glasses are also promising laser materials for high-peak-power and high-average-power lasers and short pulse generation tunable lasers, for example [10,11]. They exhibit a wider transmission range compared to silica and silicate glasses. The linear and non-linear refractive indices of the germanate and tellurite glasses are high, and these glasses also have low phonon energy, making them suitable materials for non-linear laser and amplifier applications [12]. To the best of our knowledge, no study has been conducted on the effects of radiation treatment on the optical and spectroscopic properties of Yb^3+^ doped glasses in these systems. Such study could lead to the engineering of new glasses with tailored photo-response and could be of interest when developing new glasses for space [13] or nuclear [14] applications, where radiation can severely impact the performance of the glass. Indeed, it was shown recently that radiations by electrons and protons could be used to increase the emission intensity at 1.5µm from an Er^3+^ doped phosphate glass [15].

In this context, phosphate, borosilicate, germanate, and tellurite glasses were prepared with Yb^3+^ ions and irradiated using electrons and protons. The effect of such radiation treatments on the optical and Yb^3+^ spectroscopic properties is discussed. The recovery of the properties formed during the radiation treatments is also presented. 

## 2. Materials and Methods

Yb^3+^ doped phosphate, tellurite, borosilicate, germanate, and tellurite glasses were processed with the conventional melt-quench method. The compositions and code of the investigated glasses can be found in Table 1.

The raw materials used were NaPO_3_ (Sigma, *tech*., Darmstadt, Germany) Yb_2_O_3_ (Sigma, ≥99.9%, Darmstadt, Germany), SiO_2_ (Umicore, ≥99.99%, Brussels, Belgium), H_3_BO_3_ (Sigma, ≥99.5%, Darmstadt, Germany), NaCO_3_ (Sigma, ≥99.5%, Darmstadt, Germany), CaHPO_4_·2H_2_O (Sigma, *puriss.*, Darmstadt, Germany), GeO_2_ (Sigma, ≥99.99%, Darmstadt, Germany), Ga_2_O_3_ (Sigma, ≥99.99%, Darmstadt, Germany), BaO (Sigma, ≥99.99%, Darmstadt, Germany), TiO_2_ (Sigma, ≥99.8%, Darmstadt, Germany), TeO_2_ (Sigma, ≥99%, Darmstadt, Germany) and ZnO (Sigma, ≥99.99%, Darmstadt, Germany). Ca(PO_3_)_2_ and Sr(PO_3_)_2_ compounds were synthesized from (NH_4_)_2_HPO_4_ (Sigma, ≥99.0%, Darmstadt, Germany) and CaCO_3_ (Alfa Aesar, ≥99.0%, Kandel, Germany) or SrCO_3_ (Sigma, ≥98%, Darmstadt, Germany) as explained in [16]. A total of 25–45 g glass batches were melted in quartz (phosphate glasses at 1125 °C and borosilicate glass at 1275 °C) or in Pt (germanate glass at 1600 °C and tellurite glass at 850 °C) crucible in air.” The glasses were quenched, annealed at 40 °C below their respective glass transition temperature (T_g_) for 6 h, and finally polished into disks with a thickness of ~2 mm. The polishing was performed using standard abrasive papers in successively reducing grain size with final lapping performed on 300 nm alumina suspension

The thermal properties of the glasses were obtained using differential thermal analysis (DTA) using the Netszch JUPITER F1 (NETZSCH-Gerätebau GmbH, Selb, Germany) instrument in a Pt crucible under N_2_ atmosphere and a heating rate of 10 °C/min. The T_g_ was determined as the inflection point of the endotherm obtained by taking the first derivative of the DTA curve with an accuracy of ±3 °C.

The proton irradiation was performed with a 3 MV Tandetron™ (IFIN-HH, Magurele, Romania) accelerator [17]. The incident beam was masked to a 5 × 5 mm square to provide similar irradiated area between samples. The energy was 3 MeV, and the doses were 2.5 × 10^7^, 5 × 10^7,^ and 10 × 10^7^ Gy. The penetration depth of the protons was simulated with TRIM software (SRIM-2013, by J.F. Ziegler) (http://www.srim.org, accessed on 28 March 2022).

A linear accelerator with 2 MW peak power, tuned by an EEV M5125 (Teledyne e2v Ltd., Chelmsford, UK) type magnetron operating in S-band (2992 MHz–3001 MHz) (at Accelerators Laboratory, INFLPR, Magurele, Romania), was used for the radiation treatment with electrons. The energy of the beam was 6 MeV, and the doses were 0.5 × 10^4^, 1.0 × 10^4,^ and 5.0 × 10^4^ Gy.

The density measurement was performed via the Archimedes’ method in ethanol with a measurement accuracy of ±0.02 g/cm^3^.

The absorption spectra of the glasses were measured using a spectrophotometer (UV-3600 Plus, Shimadzu) from 200 to 1700 nm. From the absorption coefficient, the absorption cross-section $\sigma_{abs}\left(\lambda \right)$ was then calculated using Equation (1).

(1) $$\sigma_{abs}\left(\lambda \right)=\frac{\ln10\;\log\left(\frac{I\left(\lambda \right)}{I_{0}}\right)}{LN}$$

where *N* is the concentration of Yb^3+^ ions per cm^3^, *L* the thickness of the sample in cm and $\log\left(\frac{I\left(\lambda \right)}{I_{0}}\right)$ the absorbance. Accuracy of measurement was ±10%.

The emission spectra were measured from 970 nm to 1200 nm using a spectrometer (iHR320, Jobin Yvon, Horiba Ltd., Kyoto, Japan) with a detector (P4631-02, Hamamatsu Photonics K.K., Hamamatsu City, Japan). A monochromatic single-mode pigtailed laser diode (CM962UF76P-10R, Oclaro Inc., San Jose, CA, USA) was used for excitation. The excitation wavelength was temperature-adjusted down to 963 nm with an FWHM of 2 nm. The emission spectra were measured from bulk samples with equal thickness to allow for the comparison of the emission intensities. The measurement resolution was 1 nm with 2 nm slits for both absorption and emission spectra measurements.

The Raman spectra were obtained using a confocal Raman microscope (InVia Qontor, Renishaw plc., Wotton-under-Edge, UK) with a 532 nm excitation, a 1200 lines/mm grating, and a 20× objective with a ~3 µm spot size. Laser power was controlled by neutral density filters for maximum signal-to-noise ratio without saturating detector.

An optical profiler (NT1100, Wyko, Veeco Instruments Inc., Plainview, NY, USA) was used to evaluate the photo-induced surface modification. 

## 3. Results and Discussion

Phosphate, borosilicate, germanate, and tellurite glasses have been the glasses of investigation in this study in order to investigate the impact of the glass composition on the photo-response to radiation treatment using electrons and protons. 

**Figure 1:**
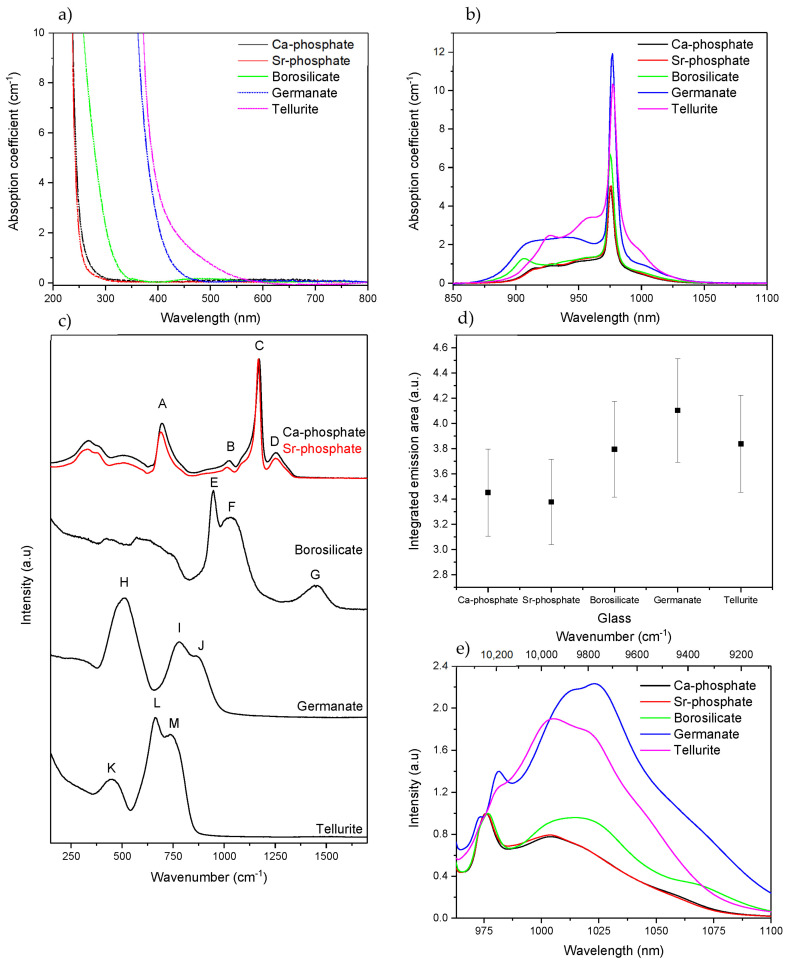
Absorption spectra (**a**,**b**), Raman spectra [see text for letter attribution] (**c**), integrated emission area (**d**) and normalized emission band of Yb^3+^ (normalization on the zero line of the ^2^F_5/2_→^2^F_7/2_ transition, see text) of the bulk glasses prior to irradiation. (**e**) (λ_exc._ = 963 nm).

Prior to discussing the effects of the radiation treatments on the optical and spectroscopic properties of the glasses, these properties of the as-prepared glasses are presented in Figure 1.

Figure 1a,b show the absorption spectra and the Yb^3+^ absorption band of the glasses, respectively. The two phosphate and the borosilicate glasses exhibit a band gap in the UV, whereas the germanate and tellurite glasses have similar optical band gaps in the blue part of the visible spectrum due to their weaker interatomic bonds. As depicted in Figure 1b, the shape, intensity, and spectral distribution of the Yb^3+^ absorption bands depend on the glass composition revealing the different local sites of the Yb^3+^ ions in the investigated glasses. The absorption cross-section at ~976 nm was calculated using Equation (1) and can be found in Table 2. 

The absorption cross-sections of the investigated glasses are similar to those reported for phosphate [18], borosilicate [19], germanate [20], and tellurite [21] glasses. One should point out that the heavy metal oxide (HMO) glasses, the germanate and tellurite glasses, have a higher absorption cross-section than the other glasses, revealing the presence of a highly asymmetric environment of the Yb^3+^ site resulting from the significant difference in the cationic field strength, i.e., the ratio of the ion charge divided by the square of the first-shell cation-oxygen distance, among the network formers surrounding Yb^3+^ [22]. 

Figure 1c presents the Raman spectra of the glasses. The Raman spectra of the phosphate glasses show four main bands, located around 690 (A), 1010 (B), 1170 (C), and 1250 cm^−1^ (D). The 690 cm^−1^ band is the result of symmetric vibration of the P-O-P bond in the metaphosphate structure [23]. The 1010 cm^−1^ is due to the symmetric vibration in P-O terminal Q^1^ units [24]. The most prominent band at 1170 cm^−1^ is caused by symmetric PO_2_ vibrations in Q^2^ units, while the band at 1250 cm^−1^ is associated with asymmetric vibrations of PO_2_ in phosphate chains [25]. Thus, according to their Raman spectra, the phosphate glasses possess a metaphosphate structure formed with infinite chains of Q^2^ units. In agreement with [26], the replacement of Ca with Sr causes the peak at 1170 cm^−1^ to shift slightly towards lower wavenumbers. Compared to the main band, the band at 1010 cm^−1^ increases in intensity while the intensity of the band at 1250 cm^−1^ decreases. These changes indicate that the network of the Sr-phosphate glass is weaker than that of the Ca-phosphate glass due to the larger ionic radius and weaker field strength of Sr^2+^ compared to Ca^2+^, as explained in [26]. 

The Raman spectrum of the borosilicate glass exhibits bands in the 400–700 cm^−1^ range that correspond to different Si-O-Si vibrations [27]. The bands in the 520–600 cm^−1^ range can be assigned to Si-O-Si links between two Q^3^ units, the band in the 590−650 cm^−1^ range to Si-O-Si linkages between two Q^2^ units, and the band at 700 cm^−1^ to bridges between two Q^1^ species [28]. The bands present in the 800–1200 cm^−1^ range are associated with the Si-O^−^ bond stretching vibrations (with O^−^ being a non-bridging oxygen (NBO)) [29]. The bands at 940 (E) and 1050 cm^−1^ (F) can be attributed to silicate tetrahedra with 2 NBOs (Q^2^ species) and one NBO (Q^3^ species), respectively [29]. The band at 1450 cm^−1^ (G) is attributable to BO_2_O^−^ triangles linked to triangular borate units, while the shoulder at 1360 cm^−1^ is attributable to BO_2_O^−^ triangles linked to [BO_4_] units, according to [30]. Thus, the structure of the borosilicate glass can be defined as a mixture of silicate and borate networks based on silicate tetrahedral, such as SiØ_3_O^−^ (Q^3^), SiØ_2_O_2_^2−^ (Q^2^), and SiØ_1_O_3_^3−^ (Q^1^) (Ø = bridging oxygen atom), with BØ_3_, BØ_2_O^−^ triangles and BØ_4_^−^ tetrahedra [31]. 

The germanate glass exhibits three main Raman bands at 500 (H), 780 (I), and 860 cm^−1^ (J). The first one corresponds to X-O-X vibrations with X = Ge or Ga [32], located in three- or four-membered GeO_4_-ring structures [33]. The band located at 780 and 860 cm^−1^ are due to Q^2^ units for Ge/Ga tetrahedra and to Q^3^ units, likely in an annular structure made from [GeO_4_] and [GaO_4_]^−^ units, respectively [32,33]. Therefore, the structure of the investigated germanate glass is likely made of a linked network containing a mixture of Q^3^ and Q^2^ units.

The Raman spectrum of the tellurite glass has three distinguishable bands at 450 (K), 660 (L), and 750 cm^−1^ (M). The 450 cm^−1^ peak is assigned to Te-O-Te link symmetric stretch in TeO_4_ or TeO_3_ polyhedra, the band at around 660 cm^−1^ to Te-O stretch in TeO_4_, and the band at 770 cm^−1^ to more distorted TeO_4_, also sometimes labeled as TeO_3+1_ [34]. Thus, the network of Te glass is formed of TeO_4_ and TeO_3+1_ units [35].

The integrated emission area and the normalized emission band of Yb^3+^ are presented in Figure 1d,e, respectively. The two phosphate glasses show a similar intensity of emission and also a similar emission band that is typical of Yb^3+^ ions located in a phosphate network [36]. Both the intensity of the emission at ~1025 nm and the bandwidth increase when Yb^3+^ is inserted in a borosilicate network rather than in a phosphate network. The intensity of the shoulder at ~1025 nm increases further when the Yb^3+^ ions are inserted in the HMO matrices due to the high disorder and the electric field surrounding the Yb^3+^ ions, as discussed earlier. Indeed, in Figure 1e, the relative intensity between the zero-line transition and the lower energy transitions highlights the effects of the reabsorption process in the overall spectral distribution. A higher absorption coefficient leads to an increased probability of reabsorption/-emission of photons emitted by the excited Yb^3+^ ions. This results in the reinforcement of the lowest emission components in parallel to the progressive quenching of the zero-line component.

The investigated glasses were subjected to radiation treatment using electrons and protons. The particle energies, doses, calculated penetration depths, and irradiation times are summarized in Table 3. 

**Figure 2:**
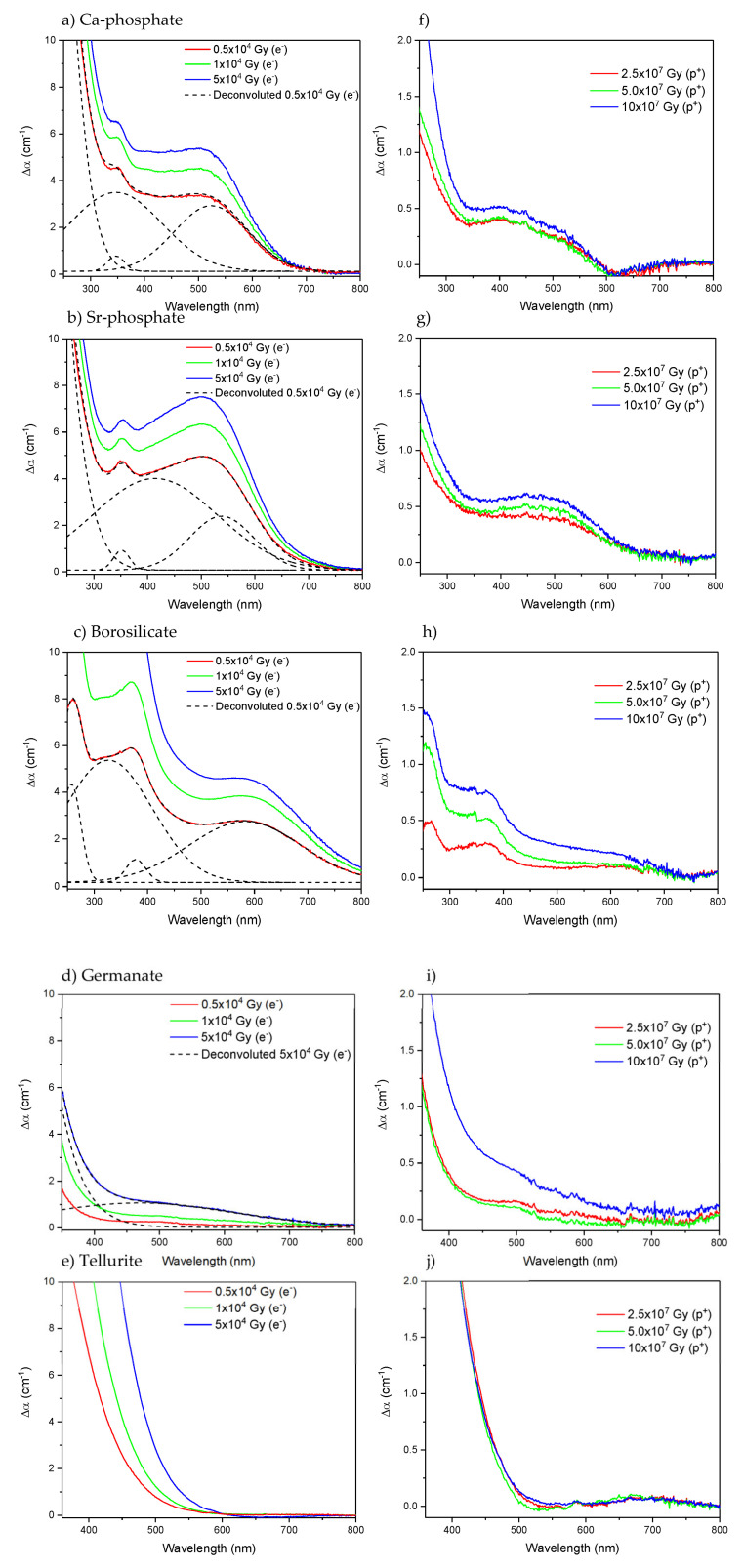
Subtracted absorption spectra prior to and after radiation treatment with electrons (**a**–**e**) and proton (**f**–**j**) of the Ca-phosphate, Sr-phosphate, borosilicate, germanate, and tellurite, respectively. (**a**–**d**) also include one deconvoluted spectrum from the figure.

No noticeable changes in the surface roughness of the glasses could be seen after both radiation treatments, even for the higher doses. No expansion nor contraction could be detected using a 3D optical profiler. However, all the investigated glasses show a darker shade after the irradiation with electrons, whereas a slight dark coloration was observed after the irradiation with protons. Those changes in the optical properties are clearly shown in Figure 2, which depicts the subtracted absorption spectra prior to and after irradiation (the absorption spectra of the glasses after irradiation can be found in Supplementary Figure S1). The radiation treatments, even at the lower doses, shift the absorption edge to longer wavelengths and result in an increase in the absorption coefficient in the 200–800 nm range, the increase of which depends not only on the doses but also on the radiation source and glass composition.

The Ca- (a, f) and Sr-phosphate (b, g) glasses show similar changes in their optical properties after irradiation, which agree with those reported in [15,37]. After the radiation treatments, new absorption bands at about 345, 420, and 530 nm appear, the intensity of which increases as the dose of electrons and protons increases. Similar absorption bands have been reported after irradiation of phosphate glasses with gamma-rays [38,39]. These bands are thought to be caused by the formation of phosphorus oxygen hole centers (POHC) [40]. The shift of the band gap to a longer wavelength might be due to the creation of other defects, such as P-related paramagnetic point defects such as the PO_3_^2−^ (phosphoryl), PO_4_^4−^ (phosphoranyl), or PO_2_^2−^ (phosphinyl), which have been reported to exhibit absorption bands at 210, 240 and 265 nm, respectively [41]. Oxygen hole centers (OHC) with an absorption band at 290 nm are also expected to form in the Ca- and Sr-phosphate glasses after the radiation treatment, according to [42]. A large increase in the absorption coefficient in the 250–550 nm range after irradiating the Sr-phosphate glass can be seen compared to the Ca-phosphate glass. This is due to the increased contribution of the POHC-related band at 420 nm and a decreased contribution of the 345 nm band. As explained previously, the main difference between the Ca-and Sr-phosphate glasses is the field strength of the Ca and Sr modifying cations. Defects, especially the POHC, appear to be produced in greater amounts in a weaker phosphate network, i.e., in the Sr-phosphate glass than in the Ca-phosphate glass, due to the difference in cationic field strength [42]. 

Figure 2c,h show the subtracted absorption spectra of borosilicate glasses after radiation treatment with electrons and protons, respectively. As observed for the phosphate glasses, new absorption bands at 260, 325, 380, and 580 nm also appear after irradiation treatment. The 260 nm and 580 nm bands can be related to the formation of non-bridging oxygen hole centers (NBOHC, ≡Si-O·) and type 2 self-trapped holes (STH_2_, ·O-Si-O·), respectively, and the 325 and 380 nm bands to oxygen-deficient centers (ODC, >Si:) [43,44,45]. Boron-related defects, namely boron bound oxygen hole centers (BOHC, ≡B-O·-Si≡) with an absorption band, are expected at around 350–450 nm based on previous studies [45]

The subtracted absorption spectra of the germanate glass after radiation treatment with electrons and protons are shown in Figure 2d,i, respectively. The radiation treatment results in a shift of the absorption edge towards longer wavelengths, likely caused by the formation of germanium-related oxygen vacancy defects (Ge-E’, ≡Ge·) at 200 nm or germanium electron centers (GEC, ≡Ge^−^-O) at 315 nm [46]. The radiation treatment results also in the appearance of a weak absorption band at ~480 nm that might be related to the reduction of Ti^4+^ into Ti^3+^ occurring during the radiation treatment, as reported in [47,48].

As opposed to the other glasses, the irradiation of tellurite glass leads only to a shift of the absorption edge to a longer wavelength, as depicted in Figure 2e, probably due to the creation of Te-related electron centers and/or hole centers which were reported to have a strong absorption band at 365 nm [49]. Similar changes in the optical properties were reported after irradiating tellurite glass with gamma-rays [50]. As depicted in Figure 2j, no changes were seen after irradiating the tellurite glasses with protons.

The differences in the optical properties after irradiation are the most distinct for the phosphate and borosilicate glasses indicating that they are the most sensitive glasses to radiation treatment, probably due to the relative lightness of their atoms (see the density of the glasses in Table 1). The HMO (germanate and tellurite) glasses appear to have the most apparent resistance against defect formation, as indicated by the small changes in their optical properties after the radiation treatment.

Yb^3+^ charge transfer (CT) -related absorption bands are a potential source for absorption bands [51,52], but, here, they cannot be resolved from the absorption spectra in Figure 2. Any such band would be expected to occur in the deep UV for all glasses [53,54,55]. Furthermore, any changes to the CT process itself would be a result of changes in the Yb^3+^ local environment, such as the defects of interest.

One should also point out that more defects are formed in the glasses after irradiation with electrons than after irradiation with protons. As explained in [15], this is due to the different penetration depths of the beams (Table 3): a greater number of defects are suspected of forming in the glass during the irradiation with electrons as the entire sample volume is irradiated while the protons barely penetrate the glasses. 

**Figure 3:**
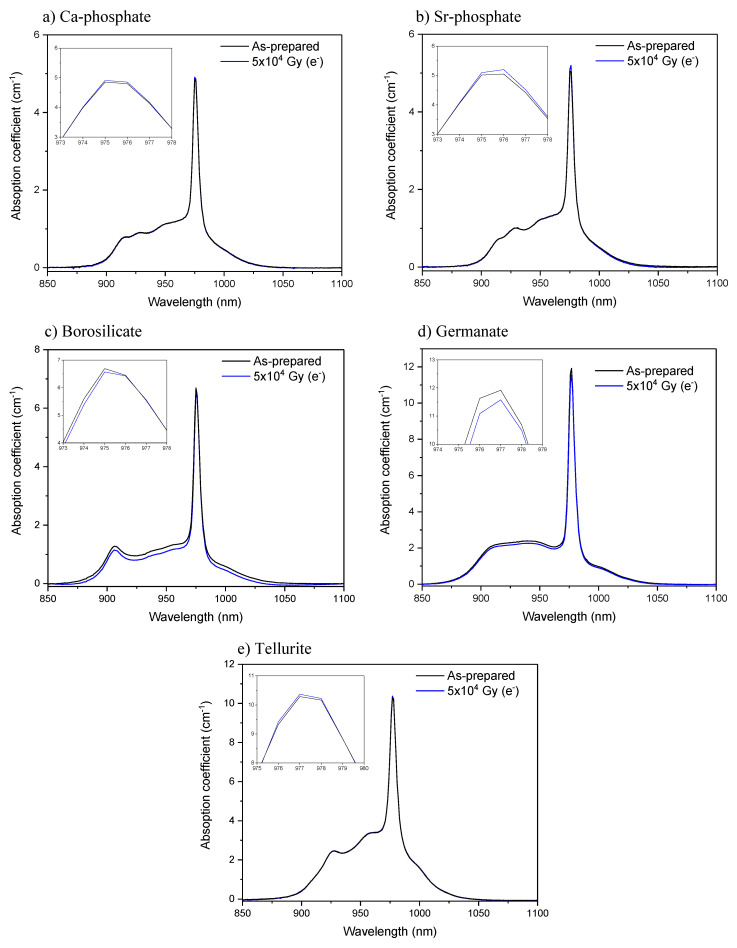
Yb^3+^ absorption band of the Ca-phosphate (**a**), Sr-phosphate (**b**), borosilicate (**c**), germanate (**d**) and tellurite (**e**) glasses prior to and after radiation treatment. Inset figures show the absorption coefficient around the peak absorbance.

**Figure 4:**
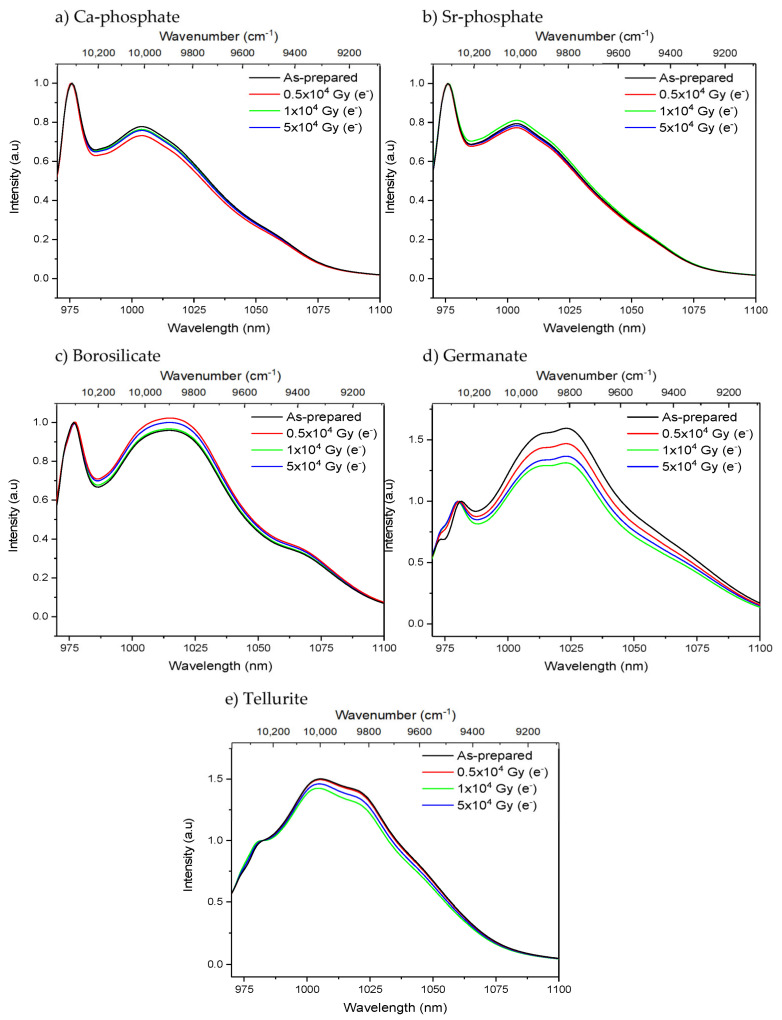
Normalized Yb^3+^ emission bands of the Ca-phosphate (**a**), Sr-phosphate (**b**), borosilicate (**c**), germanate (**d**) and tellurite (**e**) glasses prior to and after electron irradiation. The spectra are normalized at the zero-line emission band. (λ_exc._ = 963 nm).

While no noticeable changes in the Yb^3+^ absorption and emission bands of the phosphate and tellurite glasses were seen after the radiation treatments, the radiation treatment with electrons had a slight impact on the shape of the absorption and emission bands of the borosilicate and germanate glasses as depicted in Figure 3 and Figure 4, respectively. The radiation treatments have no noticeable impact on the absorption coefficient at ~975 nm of the phosphate, borosilicate, and tellurite glasses within the accuracy of the measurement (±10%), whereas a slight decrease in the absorption coefficient is suspected after irradiating the germanate glass (inset of Figure 3d). One can notice a slight change in the relative intensity of the lower energy emissions as compared to the Yb^3+^ zero-line emission in the germanate glass (Figure 4d). This reflects a reduction in the efficiency of the reabsorption process and a possible presence of additional absorbing centers or photodarkening. Thus, according to Figure 3 and Figure 4, it is possible that the irradiation of the borosilicate and germanate creates absorbing centers, the absorption of which competes with the excitation of the electron on the highest, excited component of the Yb^3+ 2^F_7/2_ excited state. It is also possible that the sites of the Yb^3+^ ions change after the radiation treatment.

**Figure 5:**
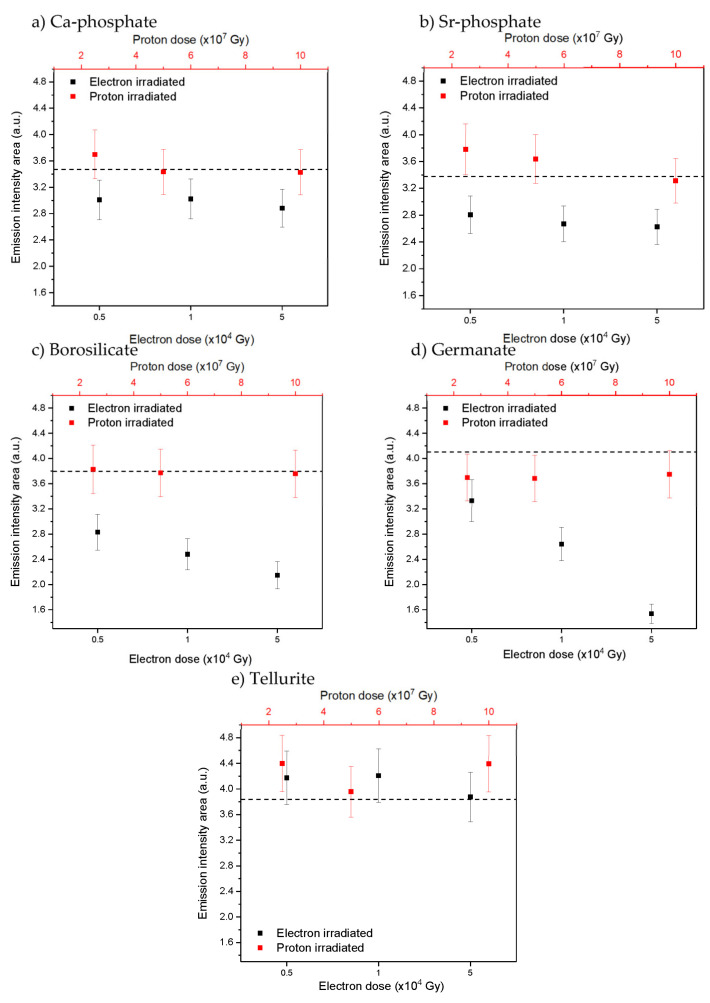
Yb^3+^ emission intensity area (λ_exc._ = 963 nm) after irradiation with varying doses for Ca-phosphate (**a**), Sr-phosphate (**b**), borosilicate (**c**), germanate (**d**) and tellurite (**e**) glasses, respectively. Dashed line shows emission intensity area for as-prepared samples, error bars ±10%.

The irradiation treatment was also found to have an impact on the overall intensity of the Yb^3+^ emission of the glasses, as illustrated in Figure 5.

Except for the tellurite glass, a decrease in the Yb^3+^ emission intensity area is seen after the electron radiation treatment in all glasses, the magnitude of which depends on the dose, while the changes induced by the proton radiation are negligible. The largest decrease in the emission intensity area after irradiation can be seen from the borosilicate and germanate glasses. As for the changes in the optical properties after the radiation treatment, a larger decrease in the Yb^3+^ emission intensity area is observed after irradiation with electrons than after irradiation with protons. Additionally, a larger decrease in the Yb^3+^ emission intensity area is observed after irradiation with electrons than after irradiation with protons. The decrease in the emission intensity area can be related to the changes in the Yb^3+^ absorption properties presented in Figure 3. Thus, it is possible that the radiation treatment changes the local environment of the Yb^3+^ and stabilizes the centers that quench the radiative de-excitation of Yb^3+^ ions partly. Alternatively, photo-ionization of the RE has been observed in [56] for Sm^3+^ in gamma-irradiated oxyfluorophosphate glass, for example, so, therefore, reduction of Yb^3+^ to Yb^2+^ must be considered. A decrease in the intensity of the Yb^3+^ emission was reported in silica glass [4] and in germanate crystals [57] after radiation treatment, confirming that photo-darkening might also occur in the investigated phosphate, borosilicate, and germanate glasses. All glasses exhibit various hole centers that could balance the charges from the reduced Yb^2+^. However, no evidence of photo-ionization of the other glass constituents was seen in the absorption spectra in Figure 2. As no noticeable changes of the Yb^3+^ spectroscopic properties in the tellurite glasses were seen after the radiation treatments, this glass appears to be the most photo-resistant against defect formation of the investigated glasses and so a promising glass for the development of novel Yb^3+^ doped fiber for radiation environments for example. 

**Figure 6:**
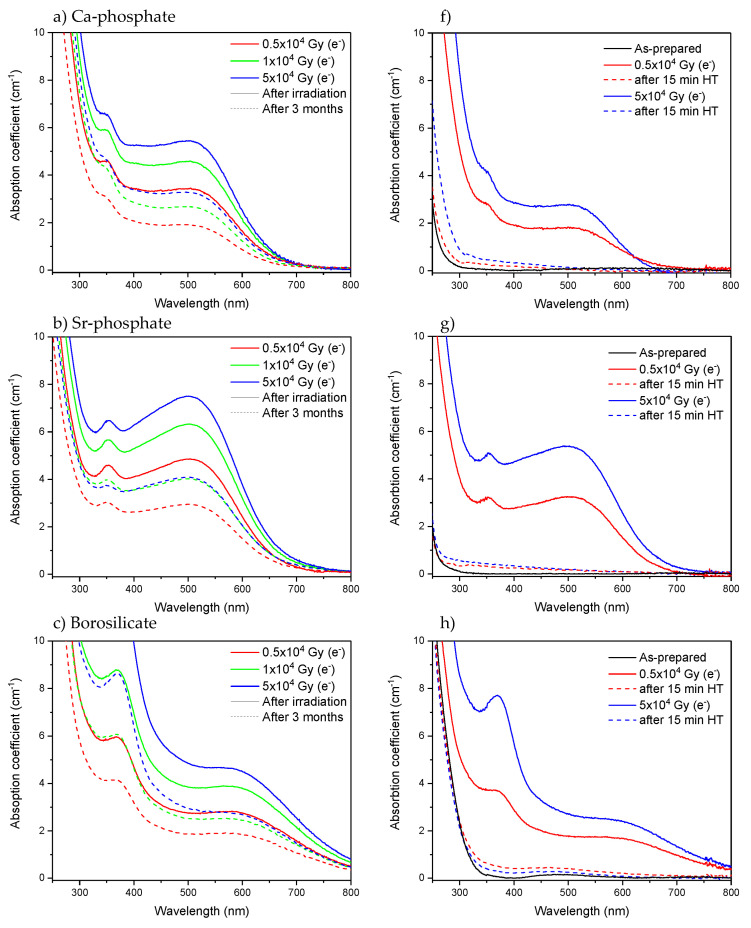

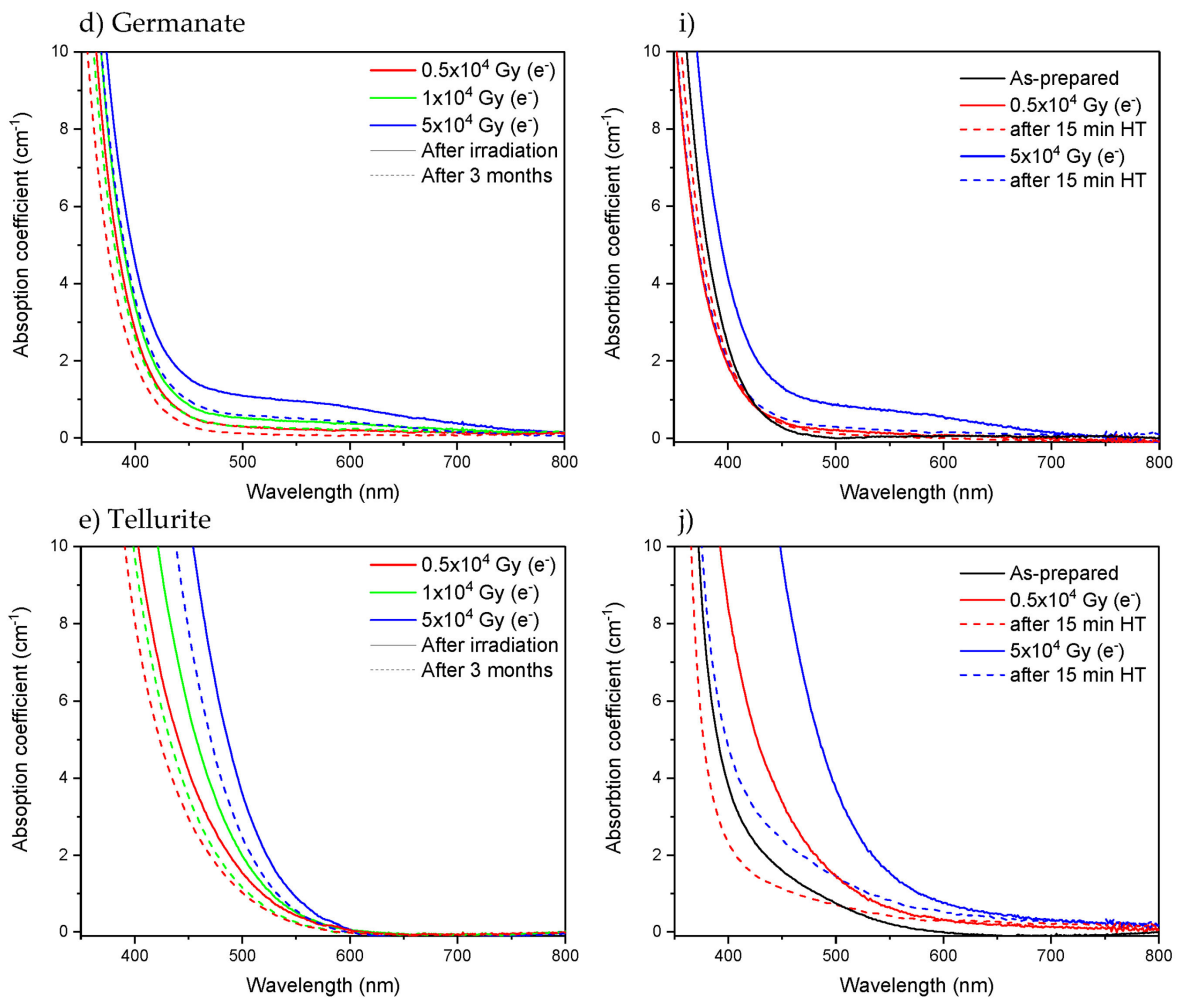
Absorption spectra of Ca-phosphate, Sr-phosphate, borosilicate, germanate, and tellurite glasses measured after irradiation using electrons (solid line), measured 3 months after the irradiation (**a**–**e**) and measured after heat treatment (HT) at T_g_ for 15 min (**f**–**j**) (dashed line).

It is well known that defects can recover over time [58,59,60,61,62]. Heat treatment could also be performed to bleach the defects [15,58,59]. Here, the absorption spectra of the glasses were measured 3 months after the radiation treatments. As shown in Figure 6a–e, a decrease of approximately 30–50% of the absorption coefficient in the 250–500 nm range is observed 3 months after the irradiation, indicating that the number of defects formed in the investigated glasses decreases over time. However, some defects still remain in the glasses after 3 months. As shown in Figure 6f–j, heat treatment of the glasses at their respective T_g_ (Table 1) for 15 min leads to a strong reduction of over 90% in the absorption coefficient in the 250–500 nm range and so in the number of defects, confirming that the defect formation is a reversible process in all the investigated glasses. Further investigation would be necessary to identify the nature of the defects and thus elucidate the mechanism behind the recovery of the defects, which is not in the scope of this study. 

**Figure 7:**
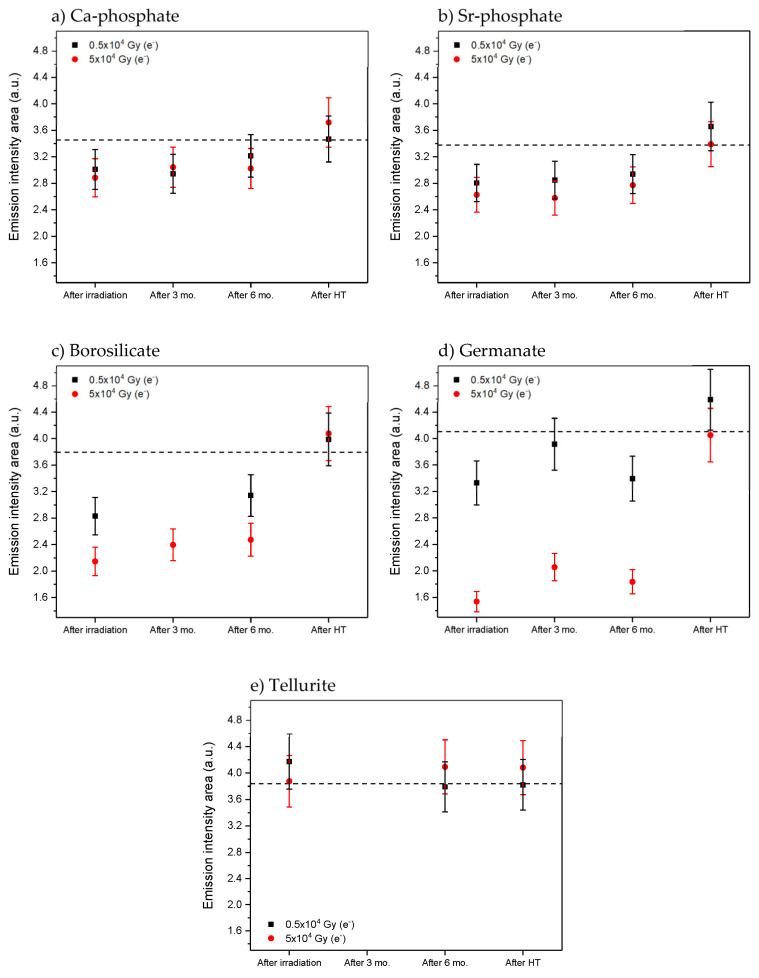
Yb^3+^ emission intensity area (λ_ex._ = 963 nm) measured after irradiation, 3 and 6 months after irradiation and also after heat treatment (HT) at T_g_ for 15 min of Ca-phosphate (**a**), Sr-phosphate (**b**), borosilicate (**c**), germanate (**d**) and tellurite (**e**) glasses, respectively. Dashed line shows emission intensity area for as-prepared sample, error bars ±10%.

As depicted in Figure 7, the Yb^3+^ emission intensity area also changes over time and after heat treatment. The heat treatment of the irradiated glasses at their respective T_g_ for 15 min results in an increase in the Yb^3+^ emission intensity area to the level of the as-prepared glasses. Similar recovery over time was also reported in irradiated phosphate glasses and silica fiber [59,60]. Recovery times are very dependent on the glass composition, defects, and radiation dose, with timescales ranging from hours [61] to several years [62].

## 4. Conclusions

In this paper, the effect of radiation treatment using electrons and protons on the optical and spectroscopic properties of a panel of Yb^3+^ doped oxide glasses was investigated. The radiation treatments result in the formation of various defects and thus changes in the optical properties of the glasses, but also in the decrease in the Yb^3+^ emission area and in the alteration of the Yb^3+^ environment in some glasses, the amplitude of these effects being dependent on the glass compositions and deposited doses. These modifications in the optical and spectroscopic properties are also more pronounced after irradiation with electrons than with protons. The borosilicate and germanate glasses were found to be the most sensitive to radiation treatment, while the tellurite glass appeared to be the least sensitive using either electrons or protons and, therefore, is a good candidate for radiation-resistant laser glasses. As mentioned for other glass systems, the color centers relax over time and can be bleached using a suitable thermal treatment of the glasses at their respective glass transition temperature, indicating that the photo-response of the glasses to irradiation with electrons and protons is a reversible process.

## Figures and Tables

**Table 1:** Nominal composition, density and glass transition temperature (T_g_) of the investigated glasses.

Code	Composition (mol-%)	ρ (g/cm^3^) ± 0.02 g/cm^3^	T_g_ (°C) ± 3 °C
Ca-phosphate	98.75 (50 P_2_O_5_-40 CaO-10 Na_2_O)-1.25 Yb_2_O_3_	2.72	476
Sr-phosphate	98.75 (50 P_2_O_5_-40 SrO-10 Na_2_O)-1.25 Yb_2_O_3_	3.14	452
Borosilicate	98.75 (26.93 SiO_2_-26.93 B_2_O_3_-22.66 Na_2_O-1.72 P_2_O5-21.76 CaO)-1.25 Yb_2_O_3_	2.75	555
Germanate	98.75 (64.6 GeO_2_-10 Ga_2_O_3_-11.4 BaO-5 TiO_2_-9 Na_2_O)-1.25 Yb_2_O_3_	4.41	613
Tellurite	98.75 (80 TeO_2_-10 ZnO-10 Na_2_O)-1.25 Yb_2_O_3_	5.24	298

**Table 2:** The absorption coefficient and cross section at ~976 nm of the investigated glasses.

Glass	Concentration of Yb^3+^ Ions (10^20^ Ions cm^−3^)	Yb^3+^ Absorption Band		
		Position of the Maximum (nm)	Absorption Coefficient at the Maximum Band Position (cm^−1^) ± 10%	Absorption Cross-Section at the Maximum Band Position (10^−20^ cm^2^) ± 10%
Ca-phosphate	4.0	976	4.8	1.2
Sr-phosphate	3.9	976	5.0	1.3
Borosilicate	6.1	976	6.7	1.1
Germanate	6.1	977	11.9	1.9
Tellurite	5.2	978	10.3	1.9

**Table 3:** The doses, penetration depths and irradiation times used for the radiation treatments.

Radiation Type	Particle Energy (MeV)	Doses (Gy)	Penetration Depth	Irradiation Time (s)
Electron (e−)	6	0.5/1.0/5.0 × 10^4^	all volume	2040–8160
Proton (p+)	3	2.5/5.0/10 × 10^7^	70–90 µm	558–6610

## Data Availability

Data is contained within the article.

## Supplementary Materials

The following supporting information can be downloaded at: https://www.mdpi.com/article/10.3390/ma15093162/s1, Figure S1: Absorption spectrum before and after irradiation treatment with electrons (**a**–**e**) and proton (**f**–**j**) irradiated Ca-phosphate, Sr-phosphate, borosilicate, germanate, and tellurite glasses respectively.
