# Supplementary material for: Response of Various Yb3+-Doped Oxide Glasses to Different Radiation Treatments
Source: Materials (Basel). 2022 Apr 27;15(9):3162. doi: 10.3390/ma15093162 (PMC9103827; doi:10.3390/ma15093162)
Supplement: Supplementary file 1 [file materials-15-03162-s001.zip › materials-1680208-supplementary.pdf]

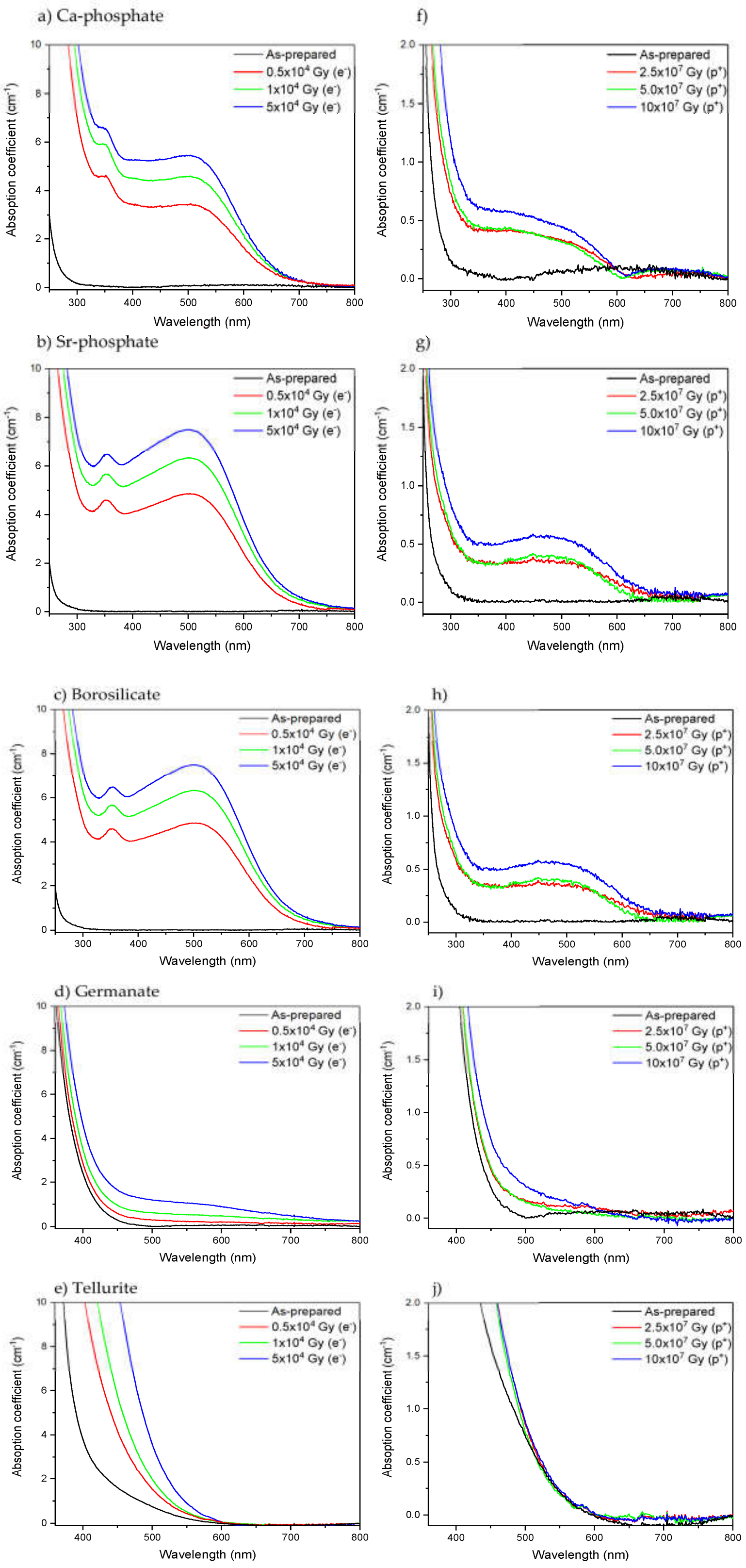

Figure S1. Absorption spectrum before and after irradiation treatment with electrons (**a**–**e**) and proton (**f**–**j**) irradiated Ca-phosphate, Sr-phosphate, borosilicate, germanate, and tellurite glasses respectively.
